# Metformin treatment prevents experimental metabolic syndrome-induced femoral bone marrow adiposity in rats

**DOI:** 10.17843/rpmesp.2024.411.13333

**Published:** 2024-03-26

**Authors:** Siro Lasalvia, Claudia Sedlinsky, León Schurman, Antonio Desmond McCarthy, Nahuel Ezequiel Wanionok

**Affiliations:** 1 Laboratory of Research on Osteopathies and Mineral Metabolism (LIOMM), Faculty of Exact Sciences, National University of La Plata. Buenos Aires, Argentina. National University of La Plata Laboratory of Research on Osteopathies and Mineral Metabolism (LIOMM) Faculty of Exact Sciences National University of La Plata Buenos Aires Argentina

**Keywords:** Metformin, metabolic syndrome, adipocytes, mesenchymal stem cells, bone tissue

## Abstract

**Objective.:**

To determine the effect of metformin (MET) treatment on adipogenic predisposition of bone marrow progenitor cells (BMPC), bone marrow adiposity and bone biomechanical properties.

**Materials and methods.:**

20 young adult male Wistar rats were sorted into four groups. Each of the groups received the following in drinking water: 100% water (C); 20% fructose (F); metformin 100 mg/kg wt/day (M); or fructose plus metformin (FM). After five weeks the animals were sacrificed. Both humeri were dissected to obtain BMPC, and both femurs were dissected to evaluate medullary adiposity (histomorphometry) and biomechanical properties (3-point bending). BMPC were cultured in vitro in adipogenic medium to evaluate RUNX2, PPAR-γ and RAGE expression by RT-PCR, lipase activity and triglyceride accumulation.

**Results.:**

The fructose-rich diet (group F) caused an increase in both triglycerides in vitro, and medullary adiposity in vivo; being partially or totally prevented by co-treatment with metformin (group FM). No differences were found in femoral biomechanical tests in vivo, nor in lipase activity and RUNX2/PPAR-γ ratio in vitro. DRF increased RAGE expression in BMPC, being prevented by co-treatment with MET.

**Conclusions.:**

Metabolic syndrome induced by a fructose-rich diet increases femoral medullary adiposity and, in part, the adipogenic predisposition of BMPC. In turn, this can be totally or partially prevented by oral co-treatment with MET.

## INTRODUCTION

Metabolic syndrome (MS) is the concurrence of three or more risk factors for heart disease, stroke, and type 2 diabetes mellitus. These factors include: high blood pressure, central obesity, low high-density lipoprotein cholesterol (HDLc), high plasma triglyceride (TG) levels, and alterations of fasting plasma glucose, with or without glucose intolerance ^1^. This syndrome is characterized by a heterogeneous composition and is currently increasing due to sedentary lifestyles and diets rich in fats and sugars ^2^. Although there is no general consensus, most studies show that MS has negative effects on bone tissue ^3^. Particularly, previous reported showed that MS was associated with a decrease in bone mineral density (BMD) of the femoral neck and lumbar spine ^4^^,^^5^, a deterioration in bone microarchitecture ^6^^,^^7^, and an increased risk of osteoporotic fracture, which in turn increases as the number of MS components increases ^8^^,^^9^. Likewise, dysglycemia and oxidative stress associated with MS promote the formation and accumulation of advanced glycation end products (AGEs), which can interact with their RAGE receptor and thus alter bone cell physiology, and/or generate aberrant crosslinks between collagen molecules that affect their biomechanical performance. We have previously demonstrated that the accumulation of AGEs in the bone extracellular matrix reduces its remodeling, with progressive accumulation of imperfections that can affect bone quality ^10^.

In previous studies, we found that experimental MS induced in Wistar rats by a diet rich in fructose (DRF) for 5 weeks, decreased the osteogenic capacity of bone marrow progenitor cells (BMPC) and altered the microarchitecture of long bones; that these changes were prevented by oral treatment with metformin. This may be due to an osteogenic/adipogenic imbalance of BMPCs towards the latter lineage, which could cause a decrease in bone formation and bone turnover, while increasing the expansion of medullary adipose tissue ^11^^-^^13^.

Metformin (MET) is an insulin-sensitizing drug, frequently used for the treatment of patients with type 2 diabetes mellitus, MS and/or glucose intolerance. Almost 20 years ago, we first demonstrated osteogenic effects of MET on cultured osteoblasts ^14^. Since then, the potential osteogenic action of MET has been studied both in preclinical models and in clinical settings. Most clinical studies reported no changes induced by MET treatment on BMD or fracture incidence ^15^^-^^17^. However, most preclinical studies found positive effects of MET on the skeletal system, increasing BMD and improving bone microarchitecture, both in normal, insulin-resistant mice and in ovariectomized rats ^18^^-^^20^. Similarly, in our laboratory we found that three weeks of MET treatment prevented the decrease in osteocyte density in trabecular bone induced by experimental MS ^13^. In a series of studies designed to evaluate the in vitro and ex vivo effects of MET on BMPC, we found that this drug promotes osteoblastogenesis and bone formation through activation of the AMPK (adenine monophosphate-activated protein kinase) pathway and increased expression of Runx2 (Runt related transcription factor 2) ^13^^,^^14^^,^^20^^-^^23^.

In this study, we assessed the effect of experimental MS induced by a DRF (with or without oral MET treatment) on bone marrow adiposity, biomechanical properties of long bones, and adipogenic predisposition of BMPC.

**KEY MESSAGES**

Motivation for the study: Most research supports a negative association between metabolic syndrome and bone health, although there is an overall lack of consensus. Therefore, there is a need for research in this area to develop a better understanding.

Main findings: Metabolic syndrome induced by a fructose-rich diet increases the adipogenic predisposition of bone marrow progenitor cells and femoral medullary adiposity in rats. Furthermore, this can be partially prevented by co-treatment with metformin.

Implications: Experimental metabolic syndrome has negative effects on bone tissue and can be prevented by oral treatment with metformin as a normoglycemic drug.

## MATERIALS AND METHODS

### Animal care and handling

We used three-month-old male Wistar rats at the beginning of the trial, which were obtained from the Faculty of Veterinary Medicine of the National University of La Plata (UNLP), Argentina. The rats were housed in cages with wood chip bedding, standard laboratory rodent chow (Asociación de Cooperativas Argentinas, Buenos Aires, Argentina) and water *ad libitum*, allowing their acclimatization under supervision for 3 days prior to the start of treatment. The housing room was maintained at 23 ± 3°C with a humidity of 30 to 70% and a 12:12h light:dark cycle. The protocol with experimental animals was approved by the Institutional Committee for the Care of Laboratory Animals of the Faculty of Exact Sciences, UNLP (Protocol # 001-00-15). In addition, the *in vivo* procedures followed the Guidelines for the Handling and Training of Laboratory Animals published by the Federation of Universities for Animal Welfare ^24^.

### Study design

Twenty clinically healthy male Wistar rats (at baseline: 12 weeks of age, body weight 300-330 g) were randomly distributed into different groups. Initially, at 12 weeks of age, half of the rats received 20% w/v fructose (Biopack, Buenos Aires, Argentina) in drinking water *ad libitum* for two weeks to induce MS. The other half of the animals continued with sterile water only. The duration of fructose administration in this study was based on our previous studies ^12^^,^^13^^)^ and others that reported that two weeks of DRF was sufficient to induce metabolic abnormalities in rats, compatible to those observed in humans with MS, such as high values of postprandial glycemia, insulinemia, HOMA insulin-resistance index, plasma triglycerides, and mesenteric adiposity ^25^^,^^26^. After two weeks of treatment, the two initial groups were again randomly divided into two subsets of 5 rats each. In these, in addition to their previous drinking water treatments, half of the animals (5 of them corresponding to the initial 20% w/v fructose and 5 to the water-only group) received 100 mg/kg/day of MET (Quimica Montpellier, Buenos Aires, Argentina) for an additional 4 weeks. Therefore, the total duration of the *in vivo* study was 6 weeks. The MET dose (100 mg/kg/day) used was higher than the dose used for humans because the plasma half-life of this drug in rats is only two minutes compared to five hours in humans, with marked pharmacodynamic differences between species ^27^. Therefore, the study design had four experimental groups: 1) control (C), 2) metformin (M), 3) fructose (F) and 4) fructose and metformin (FM).

The rats were weighed under fasting condition and anesthetized with ketamine hydrochloride/xylazine (Richmond, Buenos Aires, Argentina) once the treatments were completed. A surgical incision was made in the abdomen and 2 mL of blood was obtained from the caudal vena cava. Serum was separated in the usual manner to evaluate parameters of lipid and hydrocarbon metabolism (glycemia, triglyceridemia, cholesterolemia, HDL-cholesterol, TG/HDL ratio as a surrogate marker of insulin resistance). The anesthetized rats were then sacrificed by cervical dislocation. Mesenteric and epididymal fat were dissected; both were weighed in order to relate them to body weight and thus calculate partial and total fat indices ^28^. Then, both humeri were dissected and processed to obtain BMPC. Femurs were also removed, stripped of their musculature, fixed in 10% phosphate-buffered formalin for 48 h, decalcified in 10% EDTA and processed for bone histomorphometry as described below.

### Medullary adiposity analysis

Left femurs were embedded in kerosene, sectioned perpendicular to the longitudinal axis of the bone at a thickness of 5 µm with an RMT-20 Type Erma microtome (TechLabs, India) and stained with hematoxylin and eosin (H&E). A standardized region of interest was defined for the analysis of medullary adiposity ^21^ corresponding to the femoral head, excluding 200 µm from the growth plate in order to exclude the region corresponding to the primary spongiosa. Photomicrographs of the histological sections were taken with a Nikon Coolpix 4500 digital camera on a Nikon Eclipse E400 microscope (Nikon, Tokyo, Japan). Images were analyzed using Image J software (www.macbiophotonics.ca/imagej) with a microscope scale add-on of 40X.

### Three-point bending test

Bone biomechanical behavior was evaluated using a three-point bending test on the right femur of each animal with an electromechanical testing machine (Digimess TC500) in the midshaft. A 200 N capacity load cell (Interface, AZ, USA) was used at room temperature, with a gap length of 20 mm and a loading rate of 8 mm/min. Both, the load F (applied in the anteroposterior direction) and the displacement D until rupture, were recorded. From the obtained data, we plotted a stress-strain curve. The maximum force (Fmax) was defined as the maximum load supported by the bone before fracture. The mid-shaft structural stiffness at yield point (Fy/Dy) was calculated for each specimen from the maximum elastic deflection (Dy) and the maximum elastically supported load (Fy). Finally, toughness was defined as the amount of energy absorbed by the bone while deforming (Eabs) and was determined as the area under the stress-strain curve ^29^.

### Isolation and incubation of bone marrow progenitor cells (BMPC)

BMPCs were obtained from both humeri of all rats. The two epiphyses of each bone were sectioned, and bone marrow was obtained by washing the diaphyseal medullary canal with Dulbecco’s modified Dulbecco’s essential medium (DMEM) (Invitrogen, Buenos Aires, Argentina) under sterile conditions. The resulting cell suspensions were seeded in 25 cm^2^ culture flasks. Cells were cultured in DMEM supplemented with 10% fetal bovine serum (FBS) (Natocor, Córdoba, Argentina), penicillin (100 IU/mL) and streptomycin (100 μg/mL); and maintained at 37 °C in humidified atmosphere with 95% air and 5% CO_2_. Non-adherent cells were removed by changing the culture medium after 24 h, and thereafter two or three times per week until confluence was reached. The cell monolayer was separated by limited digestion with 0.025% trypsin (GIBCO, Invitrogen, Buenos Aires, Argentina) in 1 mM EDTA and subcultured in tissue culture plates ^22^.

Subsequently, BMPCs from all animals were cultured to confluence in 24-well plates in DMEM supplemented with 10% SFB to induce adipogenic differentiation. For this purpose, the cells were cultured for 10 additional days with medium containing: 10% DMEM-SFB plus 0.5 mmol/L 3-isobutyl-1-methylxanthine (IBMX), 1 μM dexamethasone (Decadron, Sidus, Argentina) and 200 nmol/L insulin (Lilly, Buenos Aires, Argentina). Finally, the cells were lysed with 0.1% Triton-X100 ^13^.

Intracellular triglyceride deposits and lipase activity were analyzed by means of commercial enzyme kits (Wiener, Rosario, Argentina) according to the manufacturer’s instructions. Aliquots of the same extracts were used for the determination of total cellular proteins by the Bradford technique ^13^.

Additionally, gene expression of regulators of osteogenesis (RUNX2) and adipogenesis (PPAR-γ), as well as the AGEs receptor (RAGE), was evaluated by semiquantitative polymerase chain reaction (PCR) using Moloney murine leukemia virus reverse transcriptase (RT) (Invitrogen, Argentina). Total RNA was extracted with TRIZOL reagent according to the manufacturer’s instructions (Invitrogen, Argentina) from BMPCs of all animals cultured for 10 days in adipogenic medium. Expression of mRNA for β-actin was used as housekeeping gene to normalize gene expression of RUNX2, PPARγ and RAGE. Specific primers for those phenotypic regulators were designed from the NCBI (National Center for Biotechnology Information) sequence database using CLC Genomics Workbench (QIAGEN) software (Supplementary Table 1), and were synthesized by Macrogen (Seoul, Republic of South Korea). RT-PCR products were separated by agarose gel electrophoresis with GelRed. The intensity of the bands was quantified using ImageJ software. Finally, we calculated the RUNX2/PPARγ ratio, normalized to β-actin.

**Table 1:** Body weight and adipose tissue indices of rats.

Body weight (gr)	Control	Metformin	Fructose	Fructose + metformin
	284.00 +/- 9.80	272.00 +/- 20.61	288.00 +/- 10.19	276.00 +/- 20.42
Mesenteric fat index (%)	1.36 +/- 0.12	1.53 +/- 0.09	1.94 +/- 0.10*	1.62 +/- 0.12
Epididymal fat index (%)	1.94 +/- 0.21	2.04 +/- 0.12	2.20 +/- 0.13	1.96 +/- 0.12
Total adipose index (%)	3.30 +/- 0.25	3.57 +/- 0.20	4.14 +/- 0.15*	3.58 +/- 0.13

Results are expressed as mean +/- SE (standard error). Differences: *p<0.05 vs. control.

### Statistical analysis

The Kolmogorov-Smirnov test was used to determine the Gaussian distribution, and the study of variance homogeneity was performed using Bartlett’s test. Significant differences between groups were determined by one-way analysis of variance (ANOVA), followed by Tukey’s post hoc multiple comparisons tests (GraphPad Software, San Diego, CA, USA). Values of p<0.05 were considered statistically significant.

## RESULTS

### General observations and serum profiles

All animals completed the experimental trial without presenting any adverse events. A significant increase in glycemia, triglyceridemia and TG/HDL ratio was observed in animals exposed to a DRF vs. C, p<0.05; (results not shown). Likewise, we found an increase in adiposity (visceral and total, but not epididymal) induced by DRF (p<0.05 vs. C); but there were no significant differences in body weights between the different experimental groups studied (Table 1).

### Marrow adiposity

**Figure 1:**
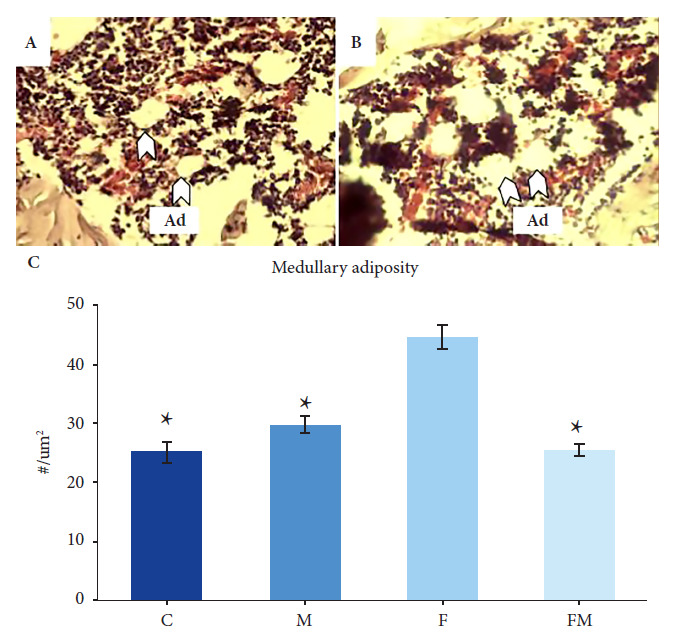
Effect of different oral treatments on femoral medullary adiposity in rats. A. Photomicrograph of histological section stained with hematoxylin-eosin, corresponding to group C (40X). B. Photomicrograph of histological section stained with hematoxylin-eosin of group F (40X). C. Effect of oral treatments on medullary adiposity

Femoral bone marrow adiposity was significantly higher in rats treated with DRF (group F) than group C animals. Likewise, co-treatment with MET (group FM) totally prevented this increase in medullary adiposity; its value being significantly lower than in the rats exposed to fructose (group F), and with no differences with respect to the Control group (Figure 1).

### Biomechanical tests

The parameters of maximum force (Fmax), maximum elastic force (Fy), structural stiffness (Fy/Dy) and tenacity (Eabs) were studied for each of the groups. No significant differences were found between groups for any of the evaluated parameters (Table 2).

**Table 2:** Femoral biomechanical parameters (3-point flexion test).

	Control	Metformin	Fructose	Fructose + metformin
Fmax (N)	111.92 +/- 9.75	109.86 +/- 4.24	102.90 +/- 7.05	112.05 +/- 6.14
Fy (N)	2.24 +/- 0.20	2.20 +/- 0.08	2.06 +/- 0.14	2.24 +/- 0.12
Fy/Dy (N/mm)	7.12 +/- 1.06	7.70 +/- 1.28	6.80 +/- 2.74	7.15 +/- 2.38
Eabs (N.mm)	50.72 +/- 3.83	47.84 +/- 1.64	47.83 +/- 4.78	46.39 +/- 4.11

Metformin (dose 100 mg/kg wt/day) in drinking water; Fructose (20% w/v) in drinking water. Results are expressed as mean +/- SE (standard error).

### Adipogenic potential of BMPCs

*Lipase activity and accumulation of triglycerides (TG)*

**Figure 2:**
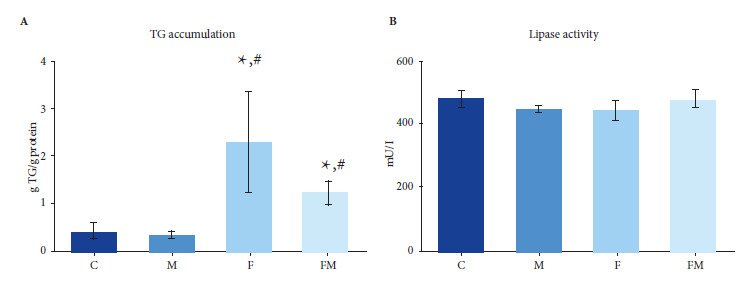
Effect of oral treatments on the in vitro adipogenic differentiation potential of BMPC. A. Triglyceride accumulation; B. Lipase activity

A higher TG content was found in THE animals treated with DRF (F group) in cell extracts obtained from BMPC cultured 10 days in adipogenic medium; this effect was partially prevented in the case of animals co-treated with MET (FM group). No significant differences in lipase activity were observed for all experimental groups (Figure 2).

### Relative gene expression of osteogenic and adipogenic regulators

**Figure 3:**
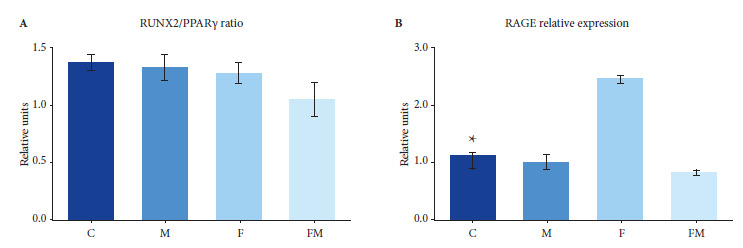
Effect of oral treatments on the expression of osteogenic and adipogenic markers. A. RUNX2/PPARγ ratio. B. Relative expression of RAGE.

BMPC obtained from animals treated with fructose and MET (FM group) showed lower expression of RUNX2 and PPARγ (normalized to β-actin) when compared to the other three experimental groups (C, F and M groups, with no differences between them). However, when we calculated the RUNX2/PPARγ ratio (more informative for assessing osteogenic versus adipogenic predisposition of BMPCs), no significant differences were found among the experimental groups (Figure 3). On the other hand, animals treated with DRF (group F) showed increased expression of RAGE (normalized to β-actin) relative to the control group, which was totally prevented by oral co-treatment with MET (group FM) (Figure 3). However, MET alone (M group) was not associated with changes in relative RAGE expression.

## DISCUSSION

In this study we have shown that experimental MS induced by a DRF increases femoral medullary adiposity and also, in part, the adipogenic predisposition of BMPCs; effects that could be mediated by an increase in the relative expression of the RAGE receptor. Likewise, we found that oral co-treatment with metformin of animals with MS prevented all these effects, showing similar values to control animals in the studied parameters. These results are compatible with those found by a large part of the studies that have been performed (although not all), which establish that MS could be detrimental to bone tissue and, additionally, that metformin promotes a greater osteogenic potential indirectly.

MS was first described more than three decades ago. Its incidence is increasing and currently affects approximately 20% of the adult population in Western countries. These characteristics make MS a real public health problem, since those who suffer from it are at high risk of associated cardiovascular disease. High fructose intake is an important inducer of the development of this syndrome, even more so considering that its consumption has increased since the introduction of high fructose corn syrup (HFCS) in the food industry. Fructose does not stimulate insulin secretion from pancreatic β-cells due to the absence of the GLUT5 transporter in these cells. In turn, fructose metabolism is not regulated by the enzyme phosphofructokinase, as is the case with the glycolytic metabolism of glucose. Excessive fructose intake can lead to oxidative stress and overproduction of inflammatory cytokines, triggering insulin resistance.

Several experimental animal models of MS have been described, which show metabolic and pathophysiological similarities with human MS. One of them consists of exposing rats to a DRF (fructose dissolved in drinking water) ^12^^,^^13^; this is the model we have used in this research. Animals exposed to a DRF showed hyperglycemia, hypertriglyceridemia, and increased visceral and total adiposity, proving the validity of the experimental model. In turn, these metabolic effects were prevented by oral co-treatment with metformin (FM group).

BMPCs are pluripotent cells, capable of differentiating into osteoblasts, adipocytes or chondrocytes. MS induces oxidative stress and a proinflammatory state in the bone marrow, even in the absence of hyperglycemia. This may affect the phenotypic fate of BMPCs, increasing their expression of PPARγ, which in turn causes a decrease in the Runx2/PPARγ ratio leading to increased adipogenic differentiation of BMPCs, and increases the number of adipocytes in the bone marrow ^10^.

In this study, by morphometric evaluation of histological sections, we were able to demonstrate a higher density of adipocytes in the femoral bone marrow of animals exposed to a DRF (group F), when compared to control animals. Likewise, by isolating BMPC and culturing them *in vitro* for 10 days in adipogenic differentiation medium, we found that cells from group F showed higher intracellular accumulation of TG (although we did not find differences for the Runx2/PPARγ gene expression ratio). Our results are in agreement with those from previous experiments in murine models of Diabetes or MS, in which we found that altered hydrocarbon metabolism caused a decrease in the *in vitro* osteogenic potential of BMPC (lower RUNX2 expression, type 1 collagen production, bone alkaline phosphatase activity, and extracellular mineralization) as well as *in vivo* alterations in bone trabecular microarchitecture (mild in MS rats, greater in diabetic rats) ^12^^,^^13^^,^^20^.

Our previous and current results indicate that alterations in glycemic metabolism could affect bone. Particularly, they could promote the adipocytic differentiation of BMPC in the bone marrow to the detriment of osteoblastogenesis, with an increase in medullary adiposity and affectation of bone architecture. One mechanism that could alter bone metabolism and bone quality is the accumulation of AGEs on type 1 collagen fibers of the extracellular matrix. On the one hand, AGEs directly alter the tensile properties of collagen (by affecting its crosslinks); and on the other hand, they can interact with the RAGE receptor, which is expressed by bone cells, inducing a decrease in bone remodeling with accumulation of imperfections ^10^. In previous studies with diabetic rats, we found that the AGE-RAGE interaction is a relevant pathway that can affect bone quality. In those studies, we observed a deterioration of trabecular bone that could be attributed to an increase in RAGE expression, induced, as a positive feedback mechanism, by an increase in tissue levels of AGEs generated by diabetic hyperglycemia. However, treatment of diabetic animals with MET improved bone architecture by preventing this AGE-induced increase in RAGE ^21^. In this research, we found an increase in the expression of the RAGE receptor in BMPC from DRF-treated rats (group F), which would be related to a decrease in the osteogenic potential of these cells. Moreover, it is consistent with the increased marrow adiposity reported by this study and with the slightly unfavorable changes in bone microarchitecture reported in our previous research ^12^^,^^13^. Although in this study we did not quantify the level of AGEs in bone, since blood glucose levels in animals with MS are much lower than those observed in diabetic rats, it is to be expected that the bone accumulation of these advanced glycation products is much lower and, therefore, no major alterations in bone microarchitecture are observed. However, this situation could be aggravated by the continued accumulation of AGEs in bone tissue over time.

In this work, in order to evaluate the integrity and biomechanical behavior of long bones, we performed three-point bending tests of the femur to its fracture point. However, we did not find significant differences between groups, which may be explained by the relatively mild effects of MS on both bone marrow adiposity (observed in this study), trabecular bone architecture (reported in our previous studies) ^12^^,^^13^, and extracellular accumulation of AGEs.

More than a decade ago, we reported for the first time the direct osteogenic effects of MET on osteoblasts in culture and BMPC, mediated by activation of AMPK ^14^^,^^22^, promoting osteoblast proliferation and, consequently, favoring bone formation and remodeling ^30^. This has been confirmed by other authors, both *in vitro* and *in vivo* in animals without disease or in models of diabetes and osteoporosis ^10^. In this study we found that, in addition to its osteogenic effects, oral treatment with MET for 4 weeks can also have anti-adipogenic effects in the bone marrow of animals with MS.

Thus, we found that MET co-treatment partially prevents the adipogenic predisposition of BMPC, and totally prevents the increase in medullary adiposity, both induced by a DRF. Although *in vitro* adipogenic differentiation studies of BMPC showed no differences between groups for relative Runx2/PPARγ gene expression; based on the results we found for medullary adiposity, as well as our previous results ^13^, it is to be expected that, the Runx2/PPARγ ratio may decrease in *in situ* BMPC of animals exposed only to DRF, (i.e., in their bone marrow microenvironment); and that this decrease is prevented *in situ* by MET in the co-treated animals (FM group).

This study has some limitations. We are aware that it would be important to perform studies verifying the Runx2/PPARγ ratio and RAGE expression of BMPC in their bone marrow niche (as well as the levels of AGEs to which these cells are exposed *in vivo*), in order to ensure that the mechanism is as proposed. Another limitation could be the measurement of the liquid intake of each animal. Although, according to our controls, the ingested volume in each of the cages was similar in all cases, but we do not have exact data on the volume of liquid consumed by each particular animal.

In conclusion, we found that experimental MS induced by a DRF increases femoral medullary adiposity and, in part, the adipogenic predisposition of BMPCs. In turn, this may be totally or partially prevented by oral co-treatment with MET. Given that DRF increases RAGE expression in BMPC, and that this effect can be prevented by co-treatment with MET, we propose the AGEs/RAGE axis as a possible relevant mechanism. We are currently conducting further studies in order to establish additional cellular and molecular mechanisms that may explain the observed effects for MET and DRF.
