# Supplementary material for: Metformin treatment prevents experimental metabolic syndrome-induced femoral bone marrow adiposity in rats
Source: Rev Peru Med Exp Salud Publica. 2024 Mar 26;41(1):28–36. doi: 10.17843/rpmesp.2024.411.13333 (PMC11149757; doi:10.17843/rpmesp.2024.411.13333)
Supplement: Supplementary material. — Available in the electronic version of the RPMESP. [file rpmesp-41-01-13333-s001.pdf]

Material suplementario

Tabla suplementaria 1. Secuencias de primers para marcadores específicos para RT-PCR

Marcador	Código banco genético	Tamaño del producto (pb)		Secuencia
**Gen housekeeping**				
**β-actina**	NM_031144.3	**345**	**Fw**	CCTTCAACACCCCAGCCAT
			**Rv**	CATAGCTCTTCTCCAGGGA
**Marcadores estudiados**				
**Runx2**	NM_001278483.1	**598**	**Fw**	GCCGGGAATGATGAGAACTA
			**Rv**	TGAGAGAGGAAGGCCAGA
**PPAR-γ**	NM_013124.3	**367**	**Fw**	CCCACCAACTTCGGAATCA
			**Rv**	AACCCTTGCATCCTTCAC
**RAGE**	NM_053336.2	**1109**	**Fw**	GTCAGAACATCACAGCC
			**Rv**	CGCTTCCTCTGACTGATT
