# Supplementary material for: Metformin treatment prevents experimental metabolic syndrome-induced femoral bone marrow adiposity in rats
Source: Rev Peru Med Exp Salud Publica. 2024 Mar 26;41(1):28–36. doi: 10.17843/rpmesp.2024.411.13333 (PMC11149757; doi:10.17843/rpmesp.2024.411.13333)
Supplement: Translation

# Translation: Tratamiento con metformina previene la adiposidad de la médula ósea femoral inducida por un síndrome metabólico experimental en ratas

**Authors:** Siro Lasalvia, Claudia Sedlinsky, León Schurman, Antonio Desmond McCarthy, Nahuel Ezequiel Wanionok

**Affiliations:** 1 Laboratorio de Investigaciones en Osteopatías y Metabolismo Mineral (LIOMM), Facultad de Ciencias Exactas, Universidad Nacional de La Plata. Buenos Aires, Argentina.

**Keywords:** Metformina, síndrome metabólico, adipocitos, células madre mesenquimales de médula ósea, tejido óseo

## Abstract

**Objetivo.:**

Determinar el efecto de un tratamiento con metformina (MET) sobre la predisposición adipogénica de células progenitoras de médula ósea (CPMO), adiposidad de la médula ósea y propiedades biomecánicas óseas.

**Materiales y métodos.:**

20 ratas Wistar machos adultos jóvenes fueron separados en cuatro grupos, recibiendo en agua de bebida: 100% agua (C); 20% de fructosa (F); metformina 100 mg/kg peso/día (M); o fructosa más metformina (FM). Tras cinco semanas se sacrificaron los animales, se diseccionaron ambos húmeros para obtener CPMO, y ambos fémures para evaluar adiposidad medular (histomorfometría) y propiedades biomecánicas (flexión a 3 puntos). Las CPMO se cultivaron *in vitro* en medio adipogénico para evaluar expresión de RUNX2, PPAR-γ y RAGE por RT-PCR, actividad de lipasa y acumulación de triglicéridos.

**Resultados.:**

La dieta rica en fructosa (grupo F) produjo un aumento tanto de triglicéridos *in vitro*, como de la adiposidad medular *in vivo*; siendo parcial o totalmente prevenido por un co-tratamiento con metformina (grupo FM). No se observaron diferencias en las pruebas biomecánicas femorales *in vivo*, ni en actividad de lipasa y relación RUNX2/PPAR-γ *in vitro*. La DRF aumentó la expresión de RAGE en CPMO, siendo prevenido por co-tratamiento con MET.

**Conclusiones.:**

El síndrome metabólico inducido por una dieta rica en fructosa aumenta la adiposidad medular femoral y, en parte, la predisposición adipogénica de las CPMO. A su vez, esto puede ser prevenido total o parcialmente por un co-tratamiento oral con MET.

## INTRODUCCIÓN

Se llama síndrome metabólico (SM) a la concurrencia de tres o más factores de riesgo para enfermedad cardíaca, accidente cerebrovascular y diabetes mellitus tipo 2. Estos factores incluyen: presión arterial elevada, obesidad central, disminución de colesterol asociado a lipoproteínas de alta densidad (HDLc), niveles elevados de triglicéridos (TG) plasmáticos y glucemia plasmática en ayunas alterada, con o sin intolerancia a la glucosa ^1^. Este síndrome se caracteriza por tener una composición heterogénea y, en la actualidad, se encuentra en franco crecimiento debido a estilos de vida sedentarios y dietas ricas en grasas y azúcares ^2^. Si bien, no existe un consenso general, gran parte de los estudios demuestran que el SM tendría efectos negativos sobre el tejido óseo ^3^. En particular, se observó que el SM se asoció con una disminución en la densidad mineral ósea (DMO) del cuello femoral y columna lumbar ^4^^,^^5^, un deterioro en la microarquitectura ósea ^6^^,^^7^, y un mayor riesgo de fractura osteoporótica, que a su vez aumenta al incrementarse el número de componentes del SM ^8^^,^^9^. Asimismo, la disglucemia y el estrés oxidativo asociados al SM, promueven la formación y acumulación de productos finales de glicación avanzada (AGE), que pueden interactuar con su receptor RAGE y así alterar la fisiología de células óseas, y/o generar entrecruzamientos aberrantes entre moléculas de colágeno que afecten su desempeño biomecánico. Hemos demostrado previamente que la acumulación de AGE en la matriz extracelular ósea reduce su remodelado, con acumulación progresiva de imperfecciones que pueden afectar la calidad del hueso ^10^.

En estudios anteriores, encontramos que el SM experimental inducido en ratas Wistar por una dieta rica en fructosa (DRF) durante 5 semanas, disminuyó la capacidad osteogénica de células progenitoras de médula ósea (CPMO) y alteró la microarquitectura de huesos largos; y que dichos cambios fueron prevenidos por un tratamiento oral con metformina. Esto podría ocurrir por un desbalance osteogénico/adipogénico de las CPMO hacia este último linaje, lo cual provocaría una disminución en la formación y el recambio óseo, a la vez que incrementaría la expansión del tejido adiposo medular ^11^^-^^13^.

Metformina (MET) es un fármaco insulino-sensibilizante, muy frecuentemente utilizado para el tratamiento de pacientes con diabetes mellitus tipo 2, SM y/o intolerancia a la glucosa. Hace casi 20 años, demostramos por primera vez efectos osteogénicos de MET sobre osteoblastos en cultivo ^14^. Desde entonces se ha investigado la potencial acción osteogénica de MET, tanto en modelos preclínicos, como en un contexto clínico. En el caso de los trabajos clínicos, en la mayoría de los mismos no se observaron cambios inducidos por el tratamiento con MET sobre la DMO o la incidencia a fracturas ^15^^-^^17^. Sin embargo, en la mayoría de los estudios preclínicos se demostraron efectos positivos de MET sobre el sistema óseo, aumentando la DMO y mejorando la microarquitectura ósea, tanto en ratones normales y resistentes a la insulina, como en ratas ovariectomizadas ^18^^-^^20^. Similarmente, en nuestro laboratorio observamos que tres semanas de tratamiento con MET previno la disminución de densidad osteocítica en hueso trabecular, inducida por un SM experimental ^13^. En una serie de estudios diseñados para evaluar los efectos *in vitro* y *ex vivo* de MET sobre CPMO, encontramos que este fármaco promueve la osteoblastogénesis y la formación ósea mediante la activación de la vía AMPK (proteína quinasa activada por monofosfato de adenina) y aumento de la expresión de Runx2 (Runt related transcription factor 2) ^13^^,^^14^^,^^20^^-^^23^.

En el presente estudio, hemos investigado el efecto del SM experimental inducido por una DRF (con o sin un tratamiento oral con MET) sobre la adiposidad de la médula ósea, las propiedades biomecánicas de huesos largos, y la predisposición adipogénica de CPMO.

**MENSAJE CLAVE**

Motivación para realizar el estudio: La mayoría de las investigaciones respaldan una asociación negativa entre el síndrome metabólico y la salud ósea, aunque existe una falta de consenso general. Por lo tanto, es necesario realizar investigaciones en esta área que permitan desarrollar un mejor conocimiento.

Principales hallazgos: El síndrome metabólico inducido por una dieta rica en fructosa incrementa la predisposición adipogénica de células progenitoras de médula ósea y la adiposidad medular femoral en ratas. Además, esto puede prevenirse parcialmente mediante un co-tratamiento con metformina.

Implicancias: El síndrome metabólico experimental posee efectos negativos sobre el tejido óseo, pudiendo ser prevenidos mediante un tratamiento oral de metformina como fármaco normoglucemiante.

## MATERIALES Y MÉTODOS

### Cuidado y manejo de animales

Utilizamos ratas Wistar machos de tres meses de edad al inicio del ensayo, las cuales fueron obtenidas en la Facultad de Veterinaria de la Universidad Nacional de La Plata (UNLP), Argentina. Las ratas se alojaron en jaulas con cama de viruta de madera, comida estándar de laboratorio para roedores (Asociación de Cooperativas Argentinas, Buenos Aires, Argentina) y agua *ad libitum,* permitiendo su aclimatación bajo supervisión durante 3 días previo al inicio de los tratamientos. La sala de alojamiento se mantuvo a 23 ± 3 °C con una humedad del 30 al 70 % y un ciclo de luz:oscuridad de 12:12 h. El protocolo con animales de experimentación fue aprobado por el Comité Institucional para el Cuidado de Animales de Laboratorio de la Facultad de Ciencias Exactas, UNLP (Protocolo # 001-00-15). Además, los procedimientos *in vivo* siguieron las Directrices para el Manejo y Entrenamiento de Animales de Laboratorio publicadas por la Federación de Universidades para el Bienestar Animal ^24^.

### Diseño del estudio

Veinte ratas Wistar machos clínicamente sanas [al inicio: 12 semanas de edad, peso corporal 300-330 g] se distribuyeron aleatoriamente en diferentes grupos. Inicialmente, a las 12 semanas de edad, la mitad de las ratas recibieron fructosa al 20 % p/v (Biopack, Buenos Aires, Argentina) en el agua potable *ad libitum* durante dos semanas para inducir SM. La otra mitad de los animales continuaron solamente con agua esterilizada. El tiempo de duración de la administración de fructosa utilizada en esta investigación se basó en nuestros estudios anteriores ^12^^,^^13^ y en otros que informaron que dos semanas de DRF fueron suficientes para inducir anomalías metabólicas en ratas, compatibles a las observadas en humanos con SM, tales como valores elevados de glucemia post-prandial, insulinemia, índice de insulino-resistencia HOMA, triglicéridos plasmáticos y adiposidad mesentérica ^25^^,^^26^. Luego de estas dos semanas de tratamiento, los dos grupos iniciales se dividieron una vez más, en forma aleatoria, en dos subconjuntos de 5 ratas cada uno. En ellos, además de sus tratamientos previos en el agua de bebida, se les administró 100 mg/kg/día de MET (Química Montpellier, Buenos Aires, Argentina) a la mitad de los animales (5 de ellos correspondientes al grupo inicial fructosa al 20 % p/v y otros 5 al de sólo agua) durante 4 semanas adicionales. Por lo tanto, la duración total del estudio *in vivo* fue de 6 semanas. La dosis de MET (100 mg/kg/día) empleada es más alta que la dosis utilizada para humanos debido a que la vida media plasmática de este fármaco en ratas es de solamente dos minutos en comparación con cinco horas en humanos, observándose marcadas diferencias farmacodinámicas entre las especies ^27^. Por lo tanto, el diseño del estudio contempló cuatro grupos experimentales: 1) control (C), 2) metformina (M), 3) fructosa (F) y 4) fructosa y metformina (FM).

Una vez finalizados los tratamientos, las ratas fueron pesadas en condición de ayuno y anestesiadas con clorhidrato de ketamina/xilazina (Richmond, Buenos Aires, Argentina). Se realizó una incisión quirúrgica en el abdomen y se obtuvieron 2 mL de sangre de la vena cava caudal. Se separó suero de la manera habitual, para evaluar parámetros del metabolismo lipídico e hidrocarbonado (glucemia, trigliceridemia, colesterolemia, HDL-colesterol, relación TG/HDL como marcador surrogante de insulino-resistencia). A continuación, las ratas anestesiadas se sacrificaron mediante dislocación cervical. Se diseccionó la grasa mesentérica y epididimal; ambas fueron pesadas con el fin de relacionarlas con el peso corporal y así calcular índices de grasas parciales y totales ^28^. Luego, ambos húmeros fueron disecados y procesados para obtener CPMO. También se extrajeron los fémures, a los cuales se le despojó la musculatura, y se fijaron en formalina tamponada con fosfato al 10 % durante 48 h, se descalcificaron en EDTA al 10% y se procesaron para histomorfometría ósea como se describe a continuación.

### Análisis de adiposidad medular

Los fémures izquierdos se incluyeron en parafina, se seccionaron perpendicularmente al eje longitudinal del hueso en un grosor de 5 µm con un micrótomo RMT-20 Tipo Erma (TechLabs, India) y se tiñeron con hematoxilina y eosina (H&E). Se definió una región de interés estandarizada para el análisis de la adiposidad medular ^21^ correspondiente a la cabeza del fémur, excluyendo 200 µm a partir del cartílago de crecimiento con la finalidad de descartar la región correspondiente a la espongiosa primaria. Se tomaron fotomicrografías de las secciones histológicas con una cámara digital Nikon Coolpix 4500 en un microscopio Eclipse E400 Nikon (Nikon, Tokio, Japón). Las imágenes se analizaron utilizando el programa Image J (www.macbiophotonics.ca/imagej) con un complemento de escala de microscopio de 40X.

### Prueba de flexión de tres puntos

Se evaluó el comportamiento biomecánico óseo mediante una prueba de flexión a tres puntos en el fémur derecho de cada animal con una máquina de prueba electromecánica (Digimess TC500) en el eje medio. Se utilizó una celda de carga de 200 N de capacidad (Interface, AZ, EE. UU.) a temperatura ambiente, con una longitud de separación de 20 mm y una velocidad de carga de 8 mm/min. Se registró la carga F (aplicada en sentido anteroposterior) y el desplazamiento D hasta la rotura. A partir de los datos obtenidos, se realizó una gráfica de la curva tensión-deformación. Se definió a la fuerza máxima (Fmáx) como la máxima carga soportada por el hueso antes de la fractura. Se calculó la rigidez estructural del eje medio en el punto de fluencia (Fy/Dy) para cada muestra a partir de la deflexión elástica máxima (Dy) y la carga máxima soportada elásticamente (Fy). Por último, la tenacidad se definió como la cantidad de energía absorbida por el hueso mientras se deformaba (Eabs) y se determinó como el área bajo la curva de tensión-deformación ^29^.

### Aislamiento e incubación de células progenitoras de médula ósea (CPMO)

Se obtuvieron las CPMO a partir de ambos húmeros de todas las ratas. Se seccionaron las dos epífisis de cada hueso, y la médula ósea se obtuvo lavando el canal medular diafisario con medio esencial modificado de Dulbecco (DMEM) (Invitrogen, Buenos Aires, Argentina) en condiciones estériles. Las suspensiones celulares resultantes se sembraron en frascos de cultivo de 25 cm^2^. Las células se cultivaron en DMEM suplementado con suero fetal bovino (SFB) al 10% (Natocor, Córdoba, Argentina), penicilina (100 UI/mL) y estreptomicina (100 μg/mL); y se mantuvieron a 37 ºC en atmósfera humidificada con 95% de aire y 5% de CO_2_. Las células no adherentes se eliminaron cambiando el medio de cultivo luego de 24 h, y a partir de entonces dos o tres veces por semana hasta alcanzar la confluencia. La monocapa celular se separó por digestión limitada con tripsina al 0,025 % (GIBCO, Invitrogen, Buenos Aires, Argentina) en EDTA 1 mM y se subcultivó en placas de cultivo de tejidos ^22^.

Posteriormente, se cultivaron CPMO de todos los animales hasta su confluencia en placas de 24 pocillos en DMEM suplementado con SFB al 10 % con la finalidad de inducir su diferenciación adipogénica. Para ello, las células se cultivaron durante 10 días adicionales con medio que contenía: DMEM-SFB al 10 % más 0,5 mmol/L de 3-isobutil-1-metilxantina (IBMX), dexametasona 1 μM (Decadron, Sidus, Argentina) e insulina 200 nmol/L (Lilly, Buenos Aires, Argentina). Finalmente, las células se lisaron con Triton-X100 al 0,1% ^13^.

Se analizaron los depósitos de triglicéridos intracelulares y la actividad lipasa mediante los respectivos kits enzimáticos comerciales (Wiener, Rosario, Argentina) de acuerdo con las instrucciones del fabricante. Se utilizaron alícuotas de los mismos extractos para la determinación de proteínas celulares totales mediante la técnica de Bradford ^13^.

Adicionalmente, se evaluó la expresión génica de reguladores de osteogénesis (RUNX2) y de adipogénesis (PPAR-γ), como también del receptor de AGEs (RAGE), a través de la reacción en cadena de la polimerasa (PCR) semicuantitativa utilizando la transcriptasa inversa (RT) del virus de la leucemia murina de Moloney (Invitrogen, Argentina). A partir de CPMO de todos los animales cultivadas durante 10 días en medio adipogénico, se extrajo el ARN total con el reactivo TRIZOL de acuerdo a las instrucciones del fabricante (Invitrogen, Argentina). La expresión de ARNm para β-actina se utilizó como gen *housekeeping* para normalizar la expresión génica de RUNX2, PPARγ y RAGE. Los cebadores específicos para dichos reguladores fenotípicos se diseñaron a partir de la base de datos de secuencias del NCBI (*National Center for Biotechnology Information*) utilizando el software CLC *Genomics Workbench* (QIAGEN) (Tabla suplementaria 1), y fueron sintetizados por Macrogen (Seúl, República de Corea del Sur). Los productos de RT-PCR se separaron mediante electroforesis en gel de agarosa con GelRed. La intensidad de las bandas se cuantificó mediante el *software* ImageJ. Finalmente, se calculó la relación RUNX2/PPARγ, normalizada a β-actina.

### Análisis estadístico

Se utilizó la prueba de Kolmogorov-Smirnov para determinar la distribución gaussiana, y el estudio de homogeneidad de varianzas se realizó mediante la prueba de Bartlett. Las diferencias significativas entre los grupos se determinaron mediante un análisis de varianza de una vía (ANOVA), seguido de las pruebas de comparaciones múltiples post hoc de Tukey (GraphPad Software, San Diego, CA, EE. UU.). Valores de p<0,05 se consideraron estadísticamente significativos.

## RESULTADOS

### Observaciones generales y perfiles séricos

La totalidad de los animales completaron el ensayo experimental sin presentar ningún tipo de incidentes adversos. Se observó un aumento significativo en la glucemia, trigliceridemia y relación TG/HDL en los animales expuestos a una DRF vs. C, p<0,05; (resultados no mostrados). Asimismo, se encontró un incremento en la adiposidad (visceral y total, pero no epididimaria) inducida por la DRF (p<0,05 vs C); pero no se registraron diferencias significativas en los pesos corporales entre los distintos grupos experimentales estudiados (Tabla 1).

**Tabla 1:** Peso corporal e índices de tejido adiposo de ratas.

Peso corporal (gr)	Control	Metformina	Fructosa	Fructosa + metformina
	284,00 +/- 9,80	272,00 +/- 20,61	288,00 +/- 10,19	276,00 +/- 20,42
Índice de grasa mesentérica (%)	1,36 +/- 0,12	1,53 +/- 0,09	1,94 +/- 0,10*	1,62 +/- 0,12
Índice de grasa epididimaria (%)	1,94 +/- 0,21	2,04 +/- 0,12	2,20 +/- 0,13	1,96 +/- 0,12
Índice adiposo total (%)	3,30 +/- 0,25	3,57 +/- 0,20	4,14 +/- 0,15*	3,58 +/- 0,13

Los resultados se expresan como la media +/- EE (error estandar). Diferencias: *p<0,05 vs Control.

### Adiposidad medular

La adiposidad de la médula ósea femoral fue significativamente mayor en las ratas tratadas con una DRF (grupo F) que en los animales del grupo C. Asimismo, el co-tratamiento con MET (grupo FM) previno totalmente este incremento en la adiposidad medular; siendo su valor significativamente menor que las ratas expuestas a fructosa (grupo F), y sin existir diferencias con respecto al grupo Control (Figura 1).

**Figura 1:** Efecto de los diferentes tratamientos orales sobre la adiposidad medular femoral de ratas. A. Microfotografía de corte histológico teñido con hematoxilina - eosina, correspondiente al grupo C (40X). B. Microfotografía de corte histológico teñido con hematoxilina - eosina del grupo F (40X). C. Efecto de los tratamientos orales sobre la adiposidad medular.

### Pruebas biomecánicas

Se estudiaron los parámetros de fuerza máxima (Fmáx), fuerza máxima elástica (Fy), rigidez estructural (Fy/Dy) y tenacidad (Eabs) para cada uno de los grupos. No se observaron diferencias significativas entre grupos, para ninguno de los parámetros evaluados (Tabla 2). 

**Tabla 2:** Parámetros biomecánicos femorales (prueba flexión a 3 puntos)

	Control	Metformina	Fructosa	Fructosa + metformina
Fmáx (N)	111,92 +/- 9,75	109,86 +/- 4,24	102,90 +/- 7,05	112,05 +/- 6,14
Fy (N)	2,24 +/- 0,20	2,20 +/- 0,08	2,06 +/- 0,14	2,24 +/- 0,12
Fy/Dy (N/mm)	7,12 +/- 1,06	7,70 +/- 1,28	6,80 +/- 2,74	7,15 +/- 2,38
Eabs (N.mm)	50,72 +/- 3,83	47,84 +/- 1,64	47,83 +/- 4,78	46,39 +/- 4,11

Metformina (dosis 100 mg/kg peso/día) en agua de bebida; Fructosa (20% p/v) en agua de bebida. Los resultados se expresan como la media +/- EE (error estandar).

### Potencial adipogénico de las CPMO

*Actividad lipasa y acumulación de triglicéridos (TG)*

En extractos celulares obtenidos a partir de CPMO cultivadas 10 días en medio adipogénico, se encontró un mayor contenido de TG en los que provenían de animales tratados con una DRF (grupo F); efecto que fue parcialmente prevenido en el caso de animales co-tratados con MET (grupo FM). No se observaron diferencias significativas en la actividad de lipasa para todos los grupos experimentales (Figura 2).

**Figura 2:** Efecto de los tratamientos orales sobre el potencial de diferenciación adipogénico in vitro de CPMO. A. Acumulación de triglicéridos; B. Actividad lipasa.

### Expresión génica relativa de reguladores osteogénicos y adipogénicos

Las CPMO obtenidas de animales co-tratados con fructosa y MET (grupo FM), mostraron una menor expresión de RUNX2 y de PPARγ (normalizada a β-actina) respecto de los otros tres grupos experimentales (grupos C, F y M, sin diferencias entre sí). Sin embargo, cuando se calculó la relación RUNX2/PPARγ (más informativa para evaluar la predisposición osteogénica versus adipogénica de las CPMO), no se encontraron diferencias significativas entre los grupos experimentales (Figura 3). Por otro lado, los animales tratados con una DRF (grupo F) mostraron una mayor expresión de RAGE (normalizada a β-actina) en relación al grupo control, lo cual fue prevenido totalmente por el co-tratamiento oral con MET (grupo FM) (Figura 3). Sin embargo, la MET por sí sola (grupo M) no se asoció con cambios en la expresión relativa de RAGE.

**Figura 3:** Efecto de los tratamientos orales sobre la expresión de marcadores osteogénicos y adipogénicos. A. Relación RUNX2/PPARγ. B. Expresión relativa de RAGE.

## DISCUSIÓN

En el presente estudio hemos demostrado que el SM experimental inducido por una DRF aumenta tanto la adiposidad medular femoral como también, en parte, la predisposición adipogénica de las CPMO; efectos que podrían ser mediados por un incremento en la expresión relativa del receptor RAGE. Asimismo, encontramos que el co-tratamiento oral con metformina de animales con SM previno todos estos efectos, mostrando valores similares a los animales control en los parámetros estudiados. Estos resultados son compatibles con los encontrados por gran parte de los estudios que se han realizado (aunque no todos), que establecen que el SM podría ser perjudicial para el tejido óseo y, adicionalmente, que la metformina promueve un mayor potencial osteogénico de manera indirecta.

El SM se describió por primera vez hace más de tres décadas. Su incidencia se encuentra en aumento, y actualmente afecta aproximadamente a un 20% de la población adulta en países occidentales. Estas características transforman al SM en un verdadero problema de salud pública, ya que quienes lo padecen tienen un riesgo elevado de enfermedad cardiovascular asociada. La elevada ingesta de fructosa constituye un importante inductor del desarrollo de este síndrome, aún más teniendo en cuenta que su consumo ha aumentado desde la introducción del jarabe de maíz con alto contenido de fructosa (JMAF) en la industria alimentaria. La fructosa no estimula la secreción de insulina de las células β pancreáticas debido a la ausencia del transportador GLUT5 en dichas células. A su vez, el metabolismo de la fructosa no se encuentra regulado por la enzima fosfofructoquinasa, como sí ocurre con el metabolismo glucolítico de la glucosa. Una ingesta excesiva de fructosa puede provocar estrés oxidativo y sobreproducción de citocinas inflamatorias, desencadenando una resistencia a la insulina.

Se han descripto varios modelos experimentales animales de SM, que muestran similitudes metabólicas y fisiopatológicas con el SM humano. Uno de ellos consiste en exponer ratas a una DRF (fructosa disuelta en el agua de bebida) ^12^^,^^13^, y es el modelo que hemos utilizado en la presente investigación. Los animales expuestos a una DRF mostraron hiperglucemia, hipertrigliceridemia y aumento de la adiposidad visceral y total, comprobando la validez del modelo experimental. A su vez, estos efectos metabólicos fueron prevenidos por un co-tratamiento oral con metformina (grupo FM).

Las CPMO son células pluripotentes, capaces de diferenciarse a osteoblastos, adipocitos o condrocitos. El SM induce estrés oxidativo y un estado proinflamatorio en la médula ósea, aun en ausencia de una hiperglucemia franca. Esto puede afectar el destino fenotípico de las CPMO, aumentando su expresión de PPARγ, lo cual a su vez genera una disminución en la relación Runx2/PPARγ que provoca una mayor diferenciación adipogénica de las CPMO, e incrementa el número de adipocitos en la médula ósea ^10^.

En el presente trabajo, por evaluación morfométrica de cortes histológicos, pudimos demostrar una mayor densidad de adipocitos en la médula ósea femoral de los animales expuestos a una DRF (grupo F), respecto de los animales control. Asimismo, al aislar CPMO y cultivarlos *in vitro* durante 10 días en un medio de diferenciación adipogénico, encontramos que las células provenientes del grupo F mostraban mayor acumulación intracelular de TG (si bien no observamos diferencias para la relación de expresión génica Runx2/PPARγ). Nuestros resultados actuales están de acuerdo con los de experimentos previos en modelos murinos de Diabetes o SM, donde encontramos que la alteración del metabolismo hidrocarbonado generó una disminución del potencial osteogénico *in vitro* de CPMO (menor expresión de RUNX2, producción de colágeno tipo 1, actividad de fosfatasa alcalina ósea y mineralización extracelular) así como alteraciones *in vivo* en la microarquitectura trabecular ósea (leves en ratas con SM, mayores en ratas diabéticas) ^12^^,^^13^^,^^20^.

Nuestros resultados previos y actuales indican que alteraciones en el metabolismo glucémico podrían afectar al hueso. En particular, promoverían en la médula ósea la diferenciación adipocítica de CPMO en desmedro de la osteoblastogénesis, con un incremento en la adiposidad medular y afectación de la arquitectura ósea. Un mecanismo que podría alterar el metabolismo y la calidad ósea, es la acumulación de AGE sobre fibras de colágeno tipo 1 de su matriz extracelular. Los AGE por un lado alteran en forma directa las propiedades tensiles del colágeno (por afectar sus entrecruzamientos); y por otro lado pueden interactuar con el receptor RAGE que es expresado por células óseas, induciendo una disminución en el remodelado del hueso con acumulación de imperfecciones ^10^. En estudios previos con ratas diabéticas encontramos que la interacción AGE-RAGE constituye una vía relevante que puede afectar la calidad ósea, en esos estudios observamos un deterioro del hueso trabecular que se pudo atribuir a un aumento en la expresión de RAGE, inducido, como mecanismo de retro-regulación positiva, por un incremento en los niveles tisulares de AGEs generados por la hiperglucemia diabética. Sin embargo, el tratamiento de animales diabéticos con MET mejoró la arquitectura ósea al prevenir este incremento de RAGE inducido por AGE ^21^. En la presente investigación observamos un aumento en la expresión del receptor RAGE en las CPMO provenientes de las ratas tratadas con DRF (grupo F), lo que estaría relacionado con una disminución en el potencial osteogénico de dichas células. Además, resulta concordante con el aumento de la adiposidad medular observado en este estudio y con los cambios levemente desfavorables en la microarquitectura ósea reportados en nuestras investigaciones anteriores ^12^^,^^13^. Si bien en el presente estudio no hemos cuantificado el nivel de AGE en el hueso, al ser mucho menores los niveles de glucemia en animales con SM respecto de los observados en ratas diabéticas, es esperable que sea mucho menor la acumulación ósea de estos productos de glicación avanzada y, por ende, no se observan grandes alteraciones sobre la microarquitectura del hueso. No obstante, esta situación podría agravarse debido a la acumulación contínua de AGE en el tejido óseo con el transcurso del tiempo.

En este trabajo, para evaluar la integridad y el comportamiento biomecánico de huesos largos, realizamos pruebas de flexión a tres puntos del fémur hasta su punto de fractura. Sin embargo, no observamos diferencias significativas entre grupos, lo que puede explicarse por los efectos relativamente leves del SM tanto sobre la adiposidad de la médula ósea (observada en este estudio), como sobre la arquitectura ósea trabecular (reportada en nuestros estudios previos) ^12^^,^^13^, y sobre la acumulación extracelular de AGE.

Hace más de una década, reportamos por primera vez efectos osteogénicos directos de MET sobre osteoblastos en cultivo y CPMO, mediado por activación de AMPK ^14^^,^^22^, promoviendo la proliferación de osteoblastos y, en consecuencia, favoreciendo la formación y el remodelado óseo ^30^. Esto ha sido confirmado por otros autores, tanto in vitro, como *in vivo* en animales sin patología o en modelos de Diabetes y osteoporosis ^10^. En el presente trabajo hemos encontrado que, además de sus efectos osteogénicos, un tratamiento oral con MET durante 4 semanas también puede tener efectos anti-adipogénicos en la médula ósea de animales con SM.

Así, encontramos que el co-tratamiento con MET previene parcialmente la predisposición adipogénica de CPMO, y previene totalmente el incremento en la adiposidad medular, ambas inducidas por una DRF. Aunque los estudios de diferenciación adipogénica *in vitro* de CPMO no mostraron diferencias entre grupos para la expresión génica relativa Runx2/PPARγ; en base a los resultados que hemos encontrado para la adiposidad medular (y a nuestros resultados anteriores ^13^, es de esperar que, en los animales expuestos sólo a la DRF, la relación Runx2/PPARγ pueda encontrarse disminuida en las CPMO *in situ* (es decir, en su microambiente de la médula ósea); y que esta disminución sea prevenida *in situ* por la MET en los animales co-tratados (grupo FM).

En relación a las limitaciones de este trabajo, somos conscientes que sería importante realizar estudios que verifiquen la relación Runx2/PPARγ y la expresión de RAGE de las CPMO en su nicho de la médula ósea (así como los niveles de AGEs a los que se encuentran expuestas estas células *in vivo*), con la finalidad de asegurar que el mecanismo es el propuesto. Asimismo, otra limitación podría ser la medición de la bebida ingerida por cada animal. Si bien, según nuestros controles, lo ingerido en cada una de las jaulas fue similar en todos los casos, no tenemos el dato exacto del volumen de líquido consumido por cada animal en particular.

En conclusión, encontramos que el SM experimental inducido por una DRF aumenta la adiposidad medular femoral y, en parte, la predisposición adipogénica de las CPMO. A su vez, esto puede ser prevenido total o parcialmente por un co-tratamiento oral con MET. Dado que la DRF aumenta la expresión de RAGE en CPMO, y que este efecto puede ser prevenido por co-tratamiento con MET, proponemos al eje AGEs/RAGE como un posible mecanismo relevante. Actualmente, nos encontramos realizando estudios adicionales para poder establecer mecanismos celulares y moleculares adicionales que puedan explicar los efectos observados para MET y DRF.
